# Supplementary material for: Assortative mating and within-spouse pair comparisons
Source: PLoS Genet. 2021 Nov 4;17(11):e1009883. doi: 10.1371/journal.pgen.1009883 (PMC8594845; doi:10.1371/journal.pgen.1009883)
Supplement: S1 Table — Results from simulation analyses investigating how the WSP model can control for confounding if spouses assort on the confounder. (DOCX) [file pgen.1009883.s001.docx]

**S1 Table** Model 1: Spousal correlations controlling for confounding

| **Degree of spousal correlation for the confounder** $\boldsymbol{E}$ | **WSP effect estimate of exposure** $\boldsymbol{X}$ **on outcome** $\boldsymbol{Y}$ **(unadjusted for** $\boldsymbol{E}$**):**  Simulation mean |
| --- | --- |
| True unconfounded estimate | 0.30 |
| 0 | 0.46 |
| 0.1 | 0.45 |
| 0.2 | 0.45 |
| 0.3 | 0.44 |
| 0.4 | 0.43 |
| 0.5 | 0.42 |
| 0.6 | 0.41 |
| 0.7 | 0.39 |
| 0.8 | 0.36 |
| 0.9 | 0.33 |
