# Supplementary material for: Assortative mating and within-spouse pair comparisons
Source: PLoS Genet. 2021 Nov 4;17(11):e1009883. doi: 10.1371/journal.pgen.1009883 (PMC8594845; doi:10.1371/journal.pgen.1009883)
Supplement: S2 Table — Results from simulation analyses investigating how the WSP model may be susceptible to collider bias induced by spousal assortment. (DOCX) [file pgen.1009883.s002.docx]

**S2 Table** Model 2: Assortment and collider bias

| **Degree of assortative mating on** $\boldsymbol{X}_{\boldsymbol{1}}$ | **Degree of assortative mating on** $\boldsymbol{X}_{\boldsymbol{2}}$ | **WSP effect estimate of exposure** $\boldsymbol{X}_{\boldsymbol{1}}$ **on outcome** $\boldsymbol{Y}$**:**  Simulation mean as proportion of true effect size |
| --- | --- | --- |
| 0 | (0, 0.1, 0.2, 0.3, 0.4, 0.5) | 1.00 |
| 0.1 | 0.1  0.2  0.3  0.4  0.5 | 0.99  0.98  0.97  0.96  0.95 |
| 0.2 | 0.1  0.2  0.3  0.4  0.5 | 0.98  0.96  0.94  0.92  0.90 |
| 0.3 | 0.1  0.2  0.3  0.4  0.5 | 0.97  0.93  0.90  0.87  0.83 |
| 0.4 | 0.1  0.2  0.3  0.4  0.5 | 0.95  0.91  0.86  0.81  0.76 |
| 0.5 | 0.1  0.2  0.3  0.4  0.5 | 0.93  0.87  0.80  0.73  0.67 |
