# Supplementary material for: Assortative mating and within-spouse pair comparisons
Source: PLoS Genet. 2021 Nov 4;17(11):e1009883. doi: 10.1371/journal.pgen.1009883 (PMC8594845; doi:10.1371/journal.pgen.1009883)
Supplement: S3 Table — A table containing summary-level phenotype information on the characteristics of the UK Biobank spouses, stratified by sex. (DOCX) [file pgen.1009883.s003.docx]

**S3 Table** Characteristics of the spouse sample (N$\leq$94,870)

| **Phenotype** | **Mean (SD)** | |
| --- | --- | --- |
|  | **Male spouses** | **Female spouses** |
| Birth year | 1950 (7.3) | 1951 (7.3) |
| Height (cm) | 175.9 (6.7) | 162.5 (6.1) |
| Educational attainment (years in full-time schooling) | 14.3 (2.3) | 14.0 (2.3) |
| Systolic blood pressure (mmHg) | 143 (18) | 139 (20) |
| Body mass index (kg/m^2^) | 27.8 (4.0) | 27.0 (5.0) |
| Coronary artery disease: cases (% of sample)^1^ | 3770 (7.9%) | 906 (1.7%) |

^1 Dichotomous trait so number of cases and % presented^
