# Supplementary material for: Assortative mating and within-spouse pair comparisons
Source: PLoS Genet. 2021 Nov 4;17(11):e1009883. doi: 10.1371/journal.pgen.1009883 (PMC8594845; doi:10.1371/journal.pgen.1009883)
Supplement: S4 Table — A table containing within-pair correlations for spouses and siblings for north-south and east-west birth coordinates as well as the first 10 principal components. (DOCX) [file pgen.1009883.s004.docx]

**S4 Table** Spouse and sibling pair correlations for birth coordinates and principal components

| **Variable** | **Spousal pair correlation (95% C.I.)** | **Sibling pair correlation**  **(95% C.I.)** |
| --- | --- | --- |
| Birth coordinate (North-South) | 0.62 (0.61, 0.62) | 0.92 (0.92, 0.93) |
| Birth coordinate (East-West) | 0.46 (0.46, 0.47) | 0.87 (0.86, 0.87) |
| PC1 | 0.16 (0.15, 0.17) | 0.94 (0.94, 0.94) |
| PC2 | 0.11 (0.10, 0.12) | 0.87 (0.87, 0.88) |
| PC3 | 0.26 (0.25, 0.27) | 0.90 (0.90, 0.91) |
| PC4 | 0.32 (0.31, 0.33) | 0.98 (0.97, 0.98) |
| PC5 | 0.32 (0.31, 0.33) | 0.97 (0.97, 0.98) |
| PC6 | 0.10 (0.10, 0.11) | 0.76 (0.75, 0.77) |
| PC7 | 0.22 (0.20, 0.23) | 0.90 (0.90, 0.91) |
| PC8 | 0.20 (0.19, 0.21) | 0.89 (0.89, 0.90) |
| PC9 | 0.30 (0.29, 0.31) | 0.91 (0.91, 0.92) |
| PC10 | 0.17 (0.16, 0.18) | 0.74 (0.73, 0.75) |
