# Supplementary material for: Assortative mating and within-spouse pair comparisons
Source: PLoS Genet. 2021 Nov 4;17(11):e1009883. doi: 10.1371/journal.pgen.1009883 (PMC8594845; doi:10.1371/journal.pgen.1009883)
Supplement: S5 Table — A table containing WSP and within-sibship shrinkage estimates from this study for height, educational attainment, BMI, SBP and alcohol consumption as well as within-sibship shrinkage estimates from an external preprint. (DOCX) [file pgen.1009883.s005.docx]

**S5 Table** Comparisons of WSP and within-sibship shrinkage estimates.

| **Phenotype** | **Shrinkage: % (95% C.I.)** | | |
| --- | --- | --- | --- |
|  | **WSP**  **(Including PC1-10)** | **Within-sibship**  **(Including PC1-10)** | **Within-sibship ***  **(Sibling GWAS)** |
| Height | 17% (14%, 20%) | 13% (8%, 18%) | 10% (8%, 12%) |
| Educational attainment | 71% (62%, 79%) | 51% (33%, 70%) | 46% (40%, 52%) |
| BMI | 16% (6%, 25%) | 5% (-12%, 22%) | 0% (-5%, 5%) |
| SBP | 0% (-8%, 7%) | 5% (-7%, 18%) | 0% (-6%, 6%) |
| Alcohol consumption | 14% (-5%, 33%) | 4% (-46%, 54%) | -3% (-28%, 22%) |

* Note that these estimates are taken from a biorXiv preprint (*Within-sibship GWAS provide improved estimates of direct genetic effects*. LJ Howe et al 2021). The shrinkage estimates are from the polygenic score analysis including genetic variants with P < 1x10^-5^.
