# Supplementary figures and images for: Assortative mating and within-spouse pair comparisons
Source: PLoS Genet. 2021 Nov 4;17(11):e1009883. doi: 10.1371/journal.pgen.1009883 (PMC8594845; doi:10.1371/journal.pgen.1009883)

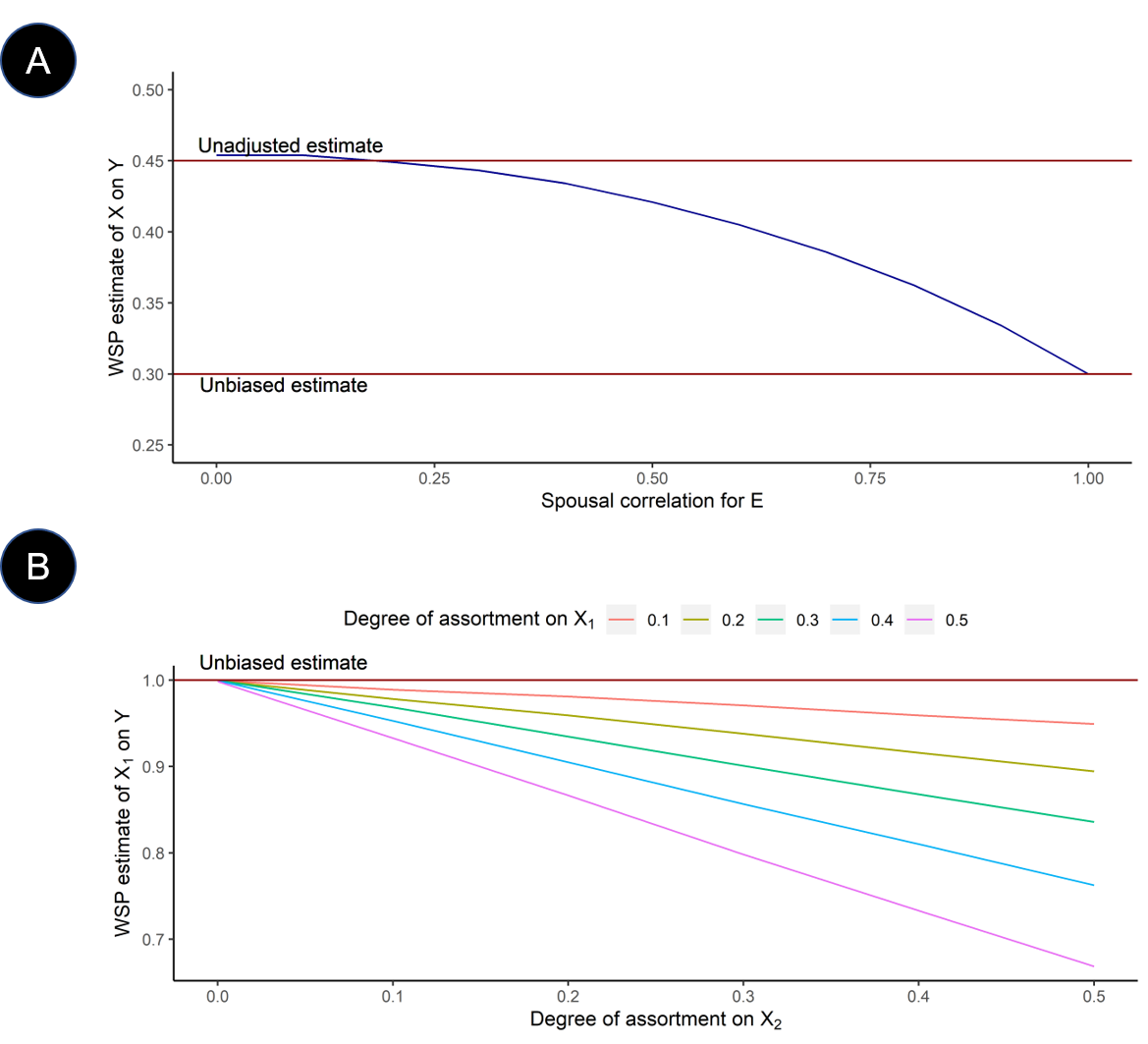

Supplement: S1 Fig — A–Simulations for model (A): Spousal correlations controlling for confounding. As the strength of spousal assortment (spousal correlation) on the confounder (E) increases, the within-spouse pair (WSP) estimate of X on Y unadjusted for E (in blue) moves from the confounded unadjusted estimate of 0.45 to the unbiased estimate of 0.30. B–Simulations for model (B): Within spouse-pair: assortment and collider bias. Spousal assortment can induce collider bias in WSP estimates. If spouses assort on two phenotypes X1 and X2 which both affect outcome Y, then the association of X1 and Y (or X2 and Y) estimated from the WSP model is a biased estimate of the causal effect of X1 on Y (or X2 on Y). This bias monotonically increases in the degree of assortment on either X1 or X2. (PNG) [file pgen.1009883.s006.png]

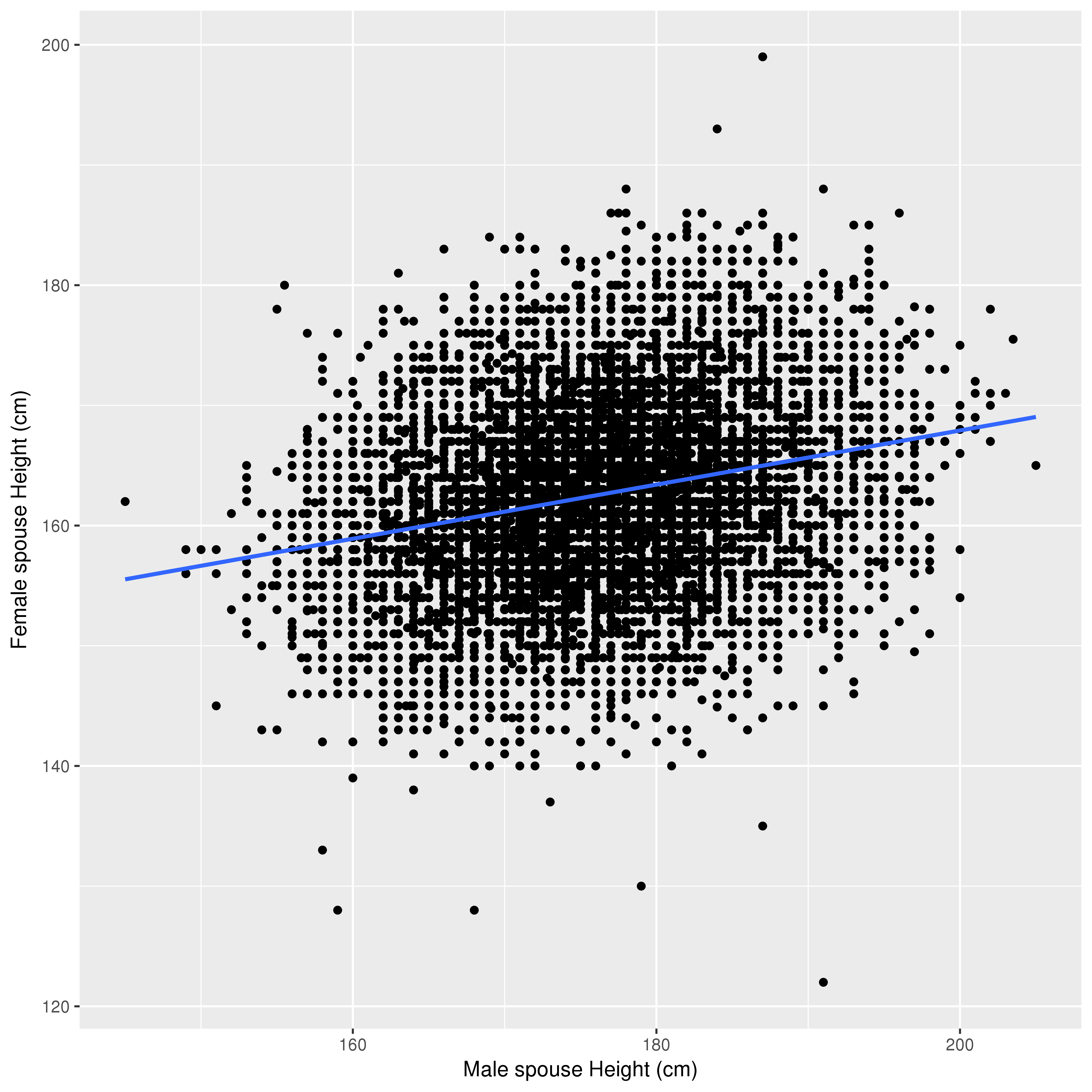

Supplement: S2 Fig — Scatter plot showing male spouse height on the X axis and female spouse height on the Y axis for each spouse-pair. (PNG) [file pgen.1009883.s007.png]

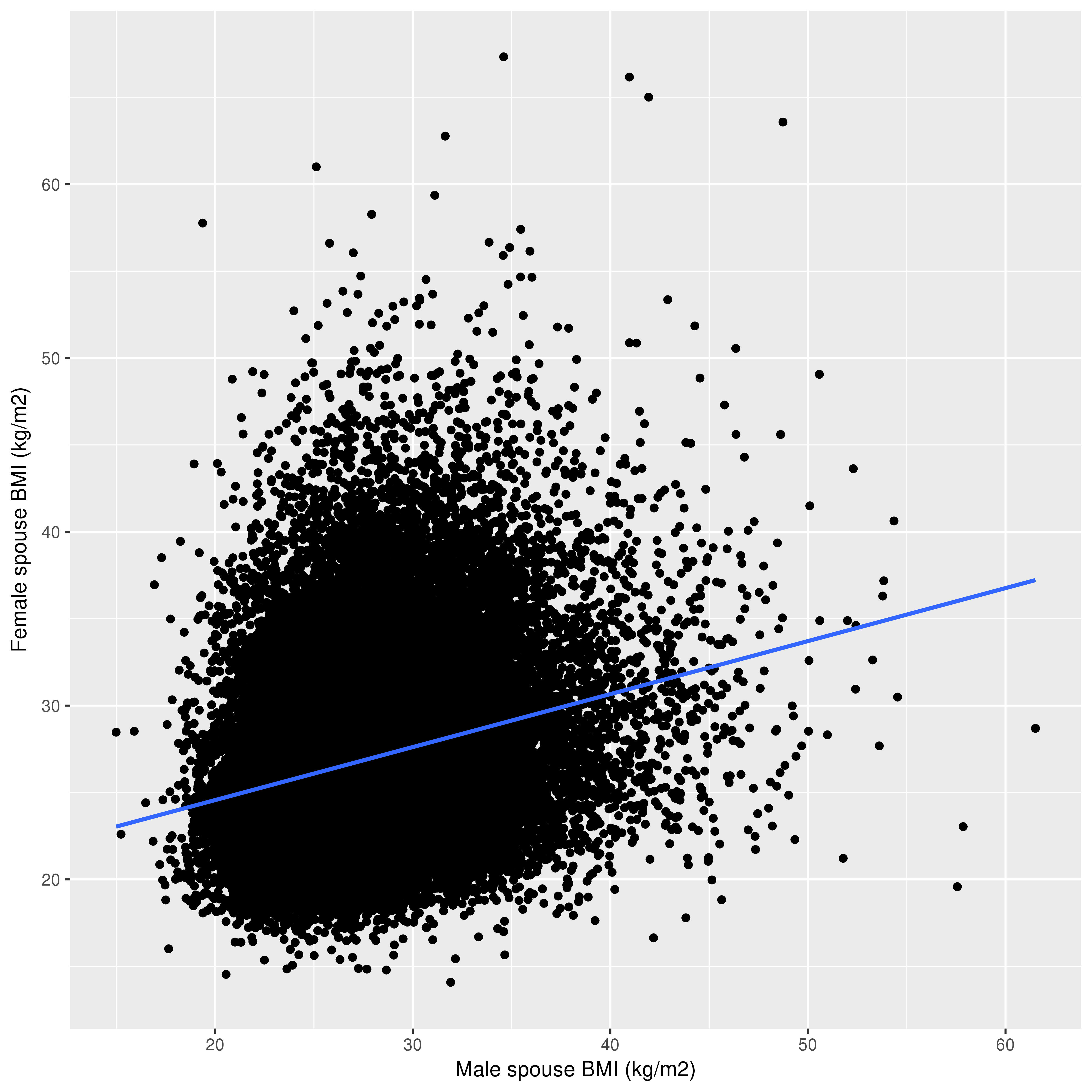

Supplement: S3 Fig — Scatter plot showing male spouse BMI on the X axis and female spouse BMI on the Y axis for each spouse-pair. (PNG) [file pgen.1009883.s008.png]

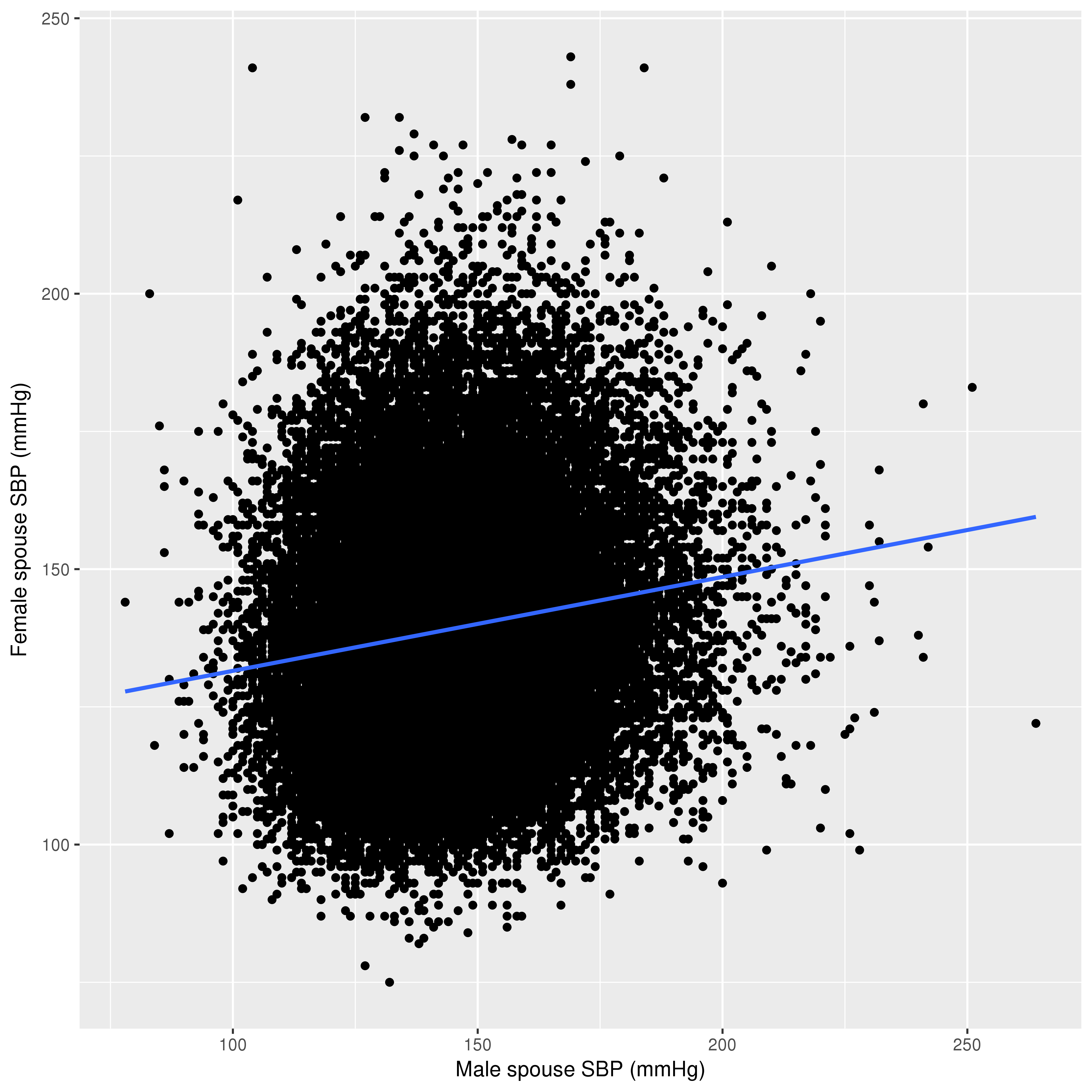

Supplement: S4 Fig — Scatter plot showing male spouse SBP on the X axis and female spouse SBP on the Y axis for each spouse-pair. (PNG) [file pgen.1009883.s009.png]
