# Supplementary material for: Assortative mating and within-spouse pair comparisons
Source: PLoS Genet. 2021 Nov 4;17(11):e1009883. doi: 10.1371/journal.pgen.1009883 (PMC8594845; doi:10.1371/journal.pgen.1009883)
Supplement: Attachment — Submitted filename: Review.docx [file pgen.1009883.s010.docx]

Howe and colleagues highlight an important study design which is often neglected in epidemiological literature: the within spouse comparison. They show that using within spouse comparison they account for some confounding effects, similar to what can be done using a within-sibling design. The advantage is that sample size for couples in biobank studies is larger than for siblings. The disadvantage is that - while siblings can be detected using genetic data - couples require registry-based or epidemiological matching which is subjected to errors.

**Comment 1**

My major comment focus on this last aspect: how sure are the authors about their definition of “couples”? They refer to a previous paper, but there is lack of validation. In particular, after the selection criteria used by the authors, 9.3% of the “couples” were of the same sex. Although this group was excluded from the analysis, this indicates that there is a substantial number of individuals potentially misclassified as couples. Probably they are just neighbors.

Few questions:

Do the author see a shrinkage in effect sizes also among the same-sex couples (which most likely are neighbors or extended family members) ?

“*If more than two individuals shared identical information, then these individuals were removed*.”. How many individuals were excluded with this criterion?

“*Closely related pairs were identified and removed using a genetic relationship matrix*”. How many individuals were additionally excluded with this criterion?

**Comment 2**

“*Within-sibship shrinkage estimates, robust to demographic and parental effects, overlapped with but were smaller than WSP shrinkage estimates*.“ I’m not sure I agree with this statement. At least looking at Table 1 there is really no difference between WSP and within-siblings estimates. If anything, within sibling shrinkage seems smaller.

**Comment 3**

Can the authors justify the use of IVW to calculate the polygenic score? A more traditional approach would be the weighted sum of betas oriented to the risk allele. It is not clear why the beta obtained from the within-couple GWAS is multiplied by the original beta from the discovery GWAS.

**Comment 4**

Would be interesting to see if restricting the random pair analysis to certain covariates reduces the shrinkage. For example, what if the random pairs are taken from the same geographical region or the same city (but not the same coordinates). At one extreme, it would be interesting to construct random pairs that are from the closest coordinates (but not the same).

**Comment 5**

The authors compare the shrinkage in PRS, but what about the effect on individual SNPs? Do they have enough power to look at the heterogeneity in shrinkage across the different SNPs?
