# Supplementary material for: Assortative mating and within-spouse pair comparisons
Source: PLoS Genet. 2021 Nov 4;17(11):e1009883. doi: 10.1371/journal.pgen.1009883 (PMC8594845; doi:10.1371/journal.pgen.1009883)
Supplement: Attachment — Submitted filename: HoweEtAl_PLOSGenet_2021.docx [file pgen.1009883.s011.docx]

In the manuscript Howe et al., titled “Assortative mating and within-spouse pair comparisons”, the authors demonstrated the potential of collider bias in within-spouse pair (WSP) study design, such that in GWAS estimating effect sizes using WSP design there is a greater degree of shrinkage compared to the gold standard within-sibship design. The paper is relatively lightweight, consisting of two or three analyses total, but I think the paper potentially brings awareness of an important topic in genetic study design. On the other hand though, despite the application in GWAS, the language of the paper seems to be tailored more towards an epidemiology audience, and might not be as familiar to human genetics community (i.e. this reviewer).

1. While I am not a domain expert on causal inference, I can appreciate the investigation into sources of bias in a GWAS. However, it is always easier for me (and I assume for the larger readership) to think about confounding bias, rather than collider bias. Thus the first set of comments I have are related to presentation.

1a. In the Introduction it would be helpful to be more explicit about how WSP design is used in genetic studies (this is submitted to a Genetics journal after all). Currently it only became more apparent how WSP (and within-sibship) designs are modeled later in the paper with the empirical example, and would require the reader to go into an even later Methods section to understand the details.

1b. It would be better to have an explicit (and more intuitive) real world example of collider bias that will help the reader appreciate the bias. The current example “association between factors which induce disease incidence are likely to be distorted in samples of only disease cases” provided little context. The spousal compatibility example is an improvement, but already assumed some level of understanding of collider bias to make sense.

1c. Similarly, Figure 1 can use some real world context. In confounding, chopsticks use or human height are commonly used example for potential confounding. It would be helpful to have something like that to illustrate collider bias.

1d. Generally, the writing can be more concrete at times. For example, “the two exposures could be correlated in the WSP model dependent on underlying model and the degree of assortment on the exposure” is a true statement, but really covered all possibilities in one sentence. Again a real world intuitive example would be helpful.

1e. I also think the direction of bias is important to be explicit when introducing collider bias with examples. To draw parallel from confounder, I can state that genetic ancestry is correlated with both the genotype (in frequency cline) and phenotype (differences in height across latitude), which results in confounding. But it is much more illustrative to say that because height value is larger in Northern Europe than in Southern Europe, alleles that are higher frequency in Northern Europe than Southern Europe will appear to be associated with increasing height if the confounder (genetic ancestry) is not adjusted. Can an example be given for collider bias, which would also provide the intuition of why there should be a shrinkage in estimates later.

2. “This suggests that population stratification is unlikely to explain the shrinkage in these estimates.” I failed to see the logic of this sentence, as it is referring to the alcohol analysis with a single, stratified, SNP. Perhaps the authors meant for this sentence to follow the previous sentence (that including PCs changed the results only a little).
3. It is interesting to note that for height, shrinkage under WSP model (even without PC) is only slightly bigger than shrinkage in within-sibship estimates. The authors attributed the additional shrinkage due to collider bias. I treat the shrinkage from within-sibship design as entirely driven by confounding due to population structure. But WSP is not otherwise modeling population structure, so the fact that the shrinkage is quite close implies that collider bias (at least for height) due to assortative mating is relatively minor, and most of the shrinkage is driven by population structure that WSP also controls?
4. On a similar line of reasoning, without explicitly modeling population structure in WSP, the fact that it appears to capture so much of the impact due to confounding from population structure is it because the analysis was done in a relatively homogeneous European subset of UKB? What would happen if the authors repeated the analysis using all of the UKB, including the non-Europeans, such that the impact due to structure should be much stronger? Would shrinkage from WSP now be smaller than shrinkage from within-sibship (because WSP is failing to control for structure completely)?
5. Discussion, paragraph 2: the authors are attributing additional shrinkage to collider. I think it should be stressed here that there is no statistically detected evidence of heterogeneity between WSP and within-sibship analysis. So it is possible that there is no collider bias in real world example. Can the authors demonstrate the existence of collider bias in real world genetic data?
6. It seems that collider bias, as investigated here due to assortative mating, is only the result when conditioning on the assortment (i.e. specifically in a WSP design). Biobank data often contains incidental spousal relationships that are not actively investigated or filtered. Can the authors discuss the impact of this more explicitly? If collider bias is only a concern in WSP designs, what implications are there in the standard GWAS design? The authors appeared wanting to discuss this on Page 12, but were quite terse.
7. Table S3 is rather uninformative. For example, mean height is given without regards to sex, which would clearly impact the mean. The table also does not provide any intuitive sense of assortment between spouses. It would be better to provide scatterplots of the phenotypes for each pair of spouse, and provide mean and sd at least by sex.
