# Supplementary material for: Assortative mating and within-spouse pair comparisons
Source: PLoS Genet. 2021 Nov 4;17(11):e1009883. doi: 10.1371/journal.pgen.1009883 (PMC8594845; doi:10.1371/journal.pgen.1009883)
Supplement: Attachment — Submitted filename: withinSpouseManuscript_reviewer_response_060721.docx [file pgen.1009883.s012.docx]

Laurence J Howe

Laurence.Howe@bristol.ac.uk

MRC Integrative Epidemiology Unit

University of Bristol

Bristol, BS8 2BN

7^th^ July 2021

David Balding

Methods Editor

*PLOS Genetics*

Dear Professor Balding,

I’m writing regarding the manuscript “*Assortative mating and within-spouse pair comparisons*”. We would like to thank the journal, editors and reviewers for their positive feedback on the manuscript and the opportunity to revise and resubmit.

In response to the editor and reviewer comments, we have made extensive changes to the manuscript, which are detailed below.

We hope that the revised manuscript is suitable for publication in *PLOS Genetics*. Thank you for considering our manuscript.

Yours sincerely,

Dr Laurence Howe on behalf of the authors

**Editors**

As you can see from the reviewers’ comments, they found the work interesting and potentially important, but what would be particularly needed is to make a better fit to a broader genetics audience. In its current format, the paper would make a better fit to an epidemiological journal. However, the reviewers give several suggestions on how to modify it to be better suited to a genetics audience with better examples from genetic analyses and examination of the behaviour of the model across a range of circumstances in simulated and/or empirical data.

Thank you for these comments, we have revised the manuscript as suggested with a focus on a genetics audience. We have integrated the suggestions of the reviewers.

**Reviewer 1**

Howe and colleagues highlight an important study design which is often neglected in epidemiological literature: the within spouse comparison. They show that using within spouse comparison they account for some confounding effects, similar to what can be done using a within-sibling design. The advantage is that sample size for couples in biobank studies is larger than for siblings. The disadvantage is that - while siblings can be detected using genetic data - couples require registry-based or epidemiological matching which is subjected to errors.

**Comment 1**

My major comment focus on this last aspect: how sure are the authors about their definition of “couples”? They refer to a previous paper, but there is lack of validation. In particular, after the selection criteria used by the authors, 9.3% of the “couples” were of the same sex. Although this group was excluded from the analysis, this indicates that there is a substantial number of individuals potentially misclassified as couples. Probably they are just neighbors.

We appreciate the author’s concerns about the definition of “couples” given that these were inferred based on questionnaire data rather than confirmed by the individuals themselves. However, in defense of the method, we note the following:

1) Similar approaches have been used by several independent research groups (see some references below) and so the approach is not solely based on a single previous manuscript and is relatively well-established in context of the UK Biobank study.

a) Robinson et al. (2017)

<https://www.nature.com/articles/s41562-016-0016>

b) Tenesa et al. (2016) <https://genomebiology.biomedcentral.com/articles/10.1186/s13059-015-0833-8>

c) Xia et al. (2020)

https://www.nature.com/articles/s41562-020-00991-9

d) Howe et al. (2019)

https://www.nature.com/articles/s41467-019-12424-x

Indeed, our approach for identifying spouses, which we first used in reference d, was based on the approach of reference a. Our approach was relatively conservative compared to reference c, which used fewer variables to match spouses and so identified around twice as many spouse-pairs.

2) The spousal phenotypic correlations for height that we estimated in our study (~0.2) is consistent with phenotypic correlations based on confirmed spouse-pairs (see for example the systematic review by Stulp et al “Assortative mating for human height: A meta‐analysis" in the *American Journal of Human Biology* (2017)). Furthermore, this is also consistent with the other studies highlighted above that used UK Biobank, regardless of their approach. While by no means definitive, this consistency is supportive of the accuracy of our derived spouse-pairs.

Few questions:

Do the author see a shrinkage in effect sizes also among the same-sex couples (which most likely are neighbors or extended family members)?

We identified around 4,000 putative same-sex couples, compared to 47,435 putative different sex couples. Some of these pairs are likely to be neighbors or extended family members, but some will likely be same-sex couples. However, we believe that same-sex couples will be less useful for comparison with the existing cross-sex analyses, because of several major practical limitations listed below.

a) The same-sex couples could be neighbours (extended family members are unlikely given our removal of pairs with IBD > 0.1) but could also be true same-sex couples. We suspect that same-sex couples are more likely to be misclassified than cross-sex couples but as the exact degree of misclassification is unknown, interpreting differences between same and cross-sex shrinkage estimates is difficult.

b) The sample size of same-sex couples (~4,000) is limited and so estimates will be far less precise than for the cross-sex couples. Tests for heterogeneity between same and cross-sex shrinkage estimates would be substantially underpowered.

c) Patterns and behaviour of assortment amongst same-sex couples may be different to assortment amongst cross-sex couples. For example, same-sex couples may have weaker assortment on height. If this were the case, then same and cross-sex shrinkage estimates for height would differ empirically.

“*If more than two individuals shared identical information, then these individuals were removed*.” How many individuals were excluded with this criterion?

We have added the relevant information to the text.

Before: “*If more than two individuals shared identical information, then these individuals were removed*.”

After: “*When more than two individuals shared identical information (observed in 18,145 instances), then these individuals were removed.”*

“*Closely related pairs were identified and removed using a genetic relationship matrix*”. How many individuals were additionally excluded with this criterion?

We have added the relevant information to the text:

Before: *“Closely related pairs were identified and removed using a genetic relationship matrix.”*

After: *“53 closely related pairs (IBD > 0.1) were identified and removed using a genetic relationship matrix”*

**Comment 2**

“*Within-sibship shrinkage estimates, robust to demographic and parental effects, overlapped with but were smaller than WSP shrinkage estimates*.“ I’m not sure I agree with this statement. At least looking at Table 1 there is really no difference between WSP and within-siblings estimates. If anything, within sibling shrinkage seems smaller.

We agree with the reviewer that the P-values for heterogeneity in Table 1 did not indicate strong evidence of heterogeneity between the WSP and within-sibship shrinkage estimates.

For comparison, we have also referenced within-sibship shrinkage estimates from an independent recent preprint (reference below) in Supplementary Table 5 (below). In this preprint, a within-sibship GWAS was conducted using a much larger sample including a consortium of 17 different studies. Within-sibship shrinkage in genetic associations was calculated using a similar method and with the additional samples, the estimates were much more precise. The consortium-based within-sibship estimates from the referenced preprint are highly consistent with the within-sibship estimates from this study. Comparing the WSP shrinkage estimates to the external multi-cohort within-sibship estimates we find stronger statistical evidence that the WSP shrinkage estimates are higher for height, BMI, and educational attainment.

Reference:

Within-sibship GWAS improve estimates of direct genetic effects

Laurence J Howe et al

bioRxiv 2021.03.05.433935; doi: https://doi.org/10.1101/2021.03.05.433935

We added the following to the manuscript on the added comparison with the external within-sibship shrinkage estimates:

Results:

“We then compared the within-sibship and WSP shrinkage estimates from this study (using UK Biobank data only) to within-sibship shrinkage estimates from a recent within-sibship GWAS of 17 cohorts [35], which included over 4x as many siblings as this study. The within-sibship shrinkage estimates from the multi-cohort GWAS were highly consistent with the within-sibship shrinkage estimates from UK Biobank only but were much more precise. The multi-cohort within-sibship shrinkage estimates were smaller than the WSP shrinkage estimates for height, BMI, and educational attainment, with non-overlapping confidence intervals, providing some evidence that WSP shrinkage is larger than within-sibship shrinkage for these phenotypes (**Supplementary Table 5**).”

**Supplementary Table 5** Comparisons of WSP and within-sibship shrinkage estimates.

| **Phenotype** | **Shrinkage: % (95% C.I.)** | | |
| --- | --- | --- | --- |
|  | **WSP**  **(Including PC1-10)** | **Within-sibship**  **(Including PC1-10)** | **Within-sibship ***  **(Sibling GWAS)** |
| Height | 17% (14%, 20%) | 13% (8%, 18%) | 10% (8%, 12%) |
| Educational attainment | 71% (62%, 79%) | 51% (33%, 70%) | 46% (40%, 52%) |
| BMI | 16% (6%, 25%) | 5% (-12%, 22%) | 0% (-5%, 5%) |
| SBP | 0% (-8%, 7%) | 5% (-7%, 18%) | 0% (-6%, 6%) |
| Alcohol consumption | 14% (-5%, 33%) | 4% (-46%, 54%) | -3% (-28%, 22%) |

* Note that these estimates are taken from a biorXiv preprint (*Within-sibship GWAS provide improved estimates of direct genetic effects*. LJ Howe et al 2021). The shrinkage estimates are from the polygenic score analysis including genetic variants with P < 1x10^-5^.

Discussion:

Before:

*Within-sibship models, which control for demographic and parental effects [30, 31], also provided evidence of shrinkage for height and educational attainment variants, consistent with previous studies [33, 34]. WSP shrinkage point estimates for height, BMI and education were larger than within-sibship shrinkage estimates although confidence intervals overlapped. The consistent evidence of shrinkage between the two models for height and education suggests that WSP models may also reduce bias in genetic association estimates.*

After:

*Within-sibship models in UK Biobank, which control for demographic and parental effects [30, 31], also provided evidence of shrinkage for height and educational attainment variants but not for BMI, consistent with previous studies [33-35]. WSP shrinkage point estimates for height, BMI and education were larger than the UK Biobank within-sibship shrinkage estimates, although confidence intervals overlapped. However, there was stronger statistical evidence that WSP shrinkage is greater than within-sibship shrinkage for height, BMI and educational attainment when using more precise shrinkage estimates from a recent within-sibship GWAS [35]. The consistent evidence of shrinkage between the two models for height and education suggests that WSP models may be removing associations relating to demography.*

Methods:

*For comparison, we also considered within-sibship shrinkage estimates from a recent within-sibship GWAS of 17 cohorts [35]. This preprint reported shrinkage for genetic variants at genome-wide significance (5x10^-8^) and a more liberal threshold (1x10^-5^) for height, BMI, educational attainment, SBP and alcohol consumption. Coronary heart disease was not analysed in this study. As the shrinkage estimates were broadly similar between the two thresholds for the 5 phenotypes in this preprint, we considered the shrinkage estimates from the liberal threshold.*

We have also made the following changes to the abstract to more accurately report our empirical findings:

Before:

*“Within-sibship shrinkage estimates, robust to demographic and parental effects, overlapped with but were smaller than WSP shrinkage estimates*.”

After:

*“Analyses using UK Biobank spouse pairs found that WSP genetic association estimates were attenuated compared to estimates from random pairs for height, educational attainment, and BMI variants. Within-sibling pair genetic association estimates, robust to demographic and parental effects, were also attenuated compared to random pair estimates for height and educational attainment, but not for BMI.”*

**Comment 3**

Can the authors justify the use of IVW to calculate the polygenic score? A more traditional approach would be the weighted sum of betas oriented to the risk allele. It is not clear why the beta obtained from the within-couple GWAS is multiplied by the original beta from the discovery GWAS.

The IVW approach is a summary-based approximation of constructing a weighted polygenic score in a sample using individual-level data and then computing associations between the polygenic score and a phenotype in that sample. The rationale behind the weighting of variants by their beta value from the discovery GWAS is to model the effect sizes of the variants in the score rather than using an unweighted score. The WSP and within-sibship shrinkage estimates are then derived using the ratio of the two polygenic score-phenotype association estimates (i.e. in the random pair and in the WSP or sibling model).

References containing the equations for this summary-based approach are:

Palla, L.and Dudbridge, F. (2015)

A fast method that uses polygenic scores to estimate the variance explained by genome-wide marker panels and the proportion of variants affecting a trait.

*American Journal of Human Genetics.*

Burgess S., Butterworth A., Thompson S.G. (2013)

Mendelian randomization analysis with multiple genetic variants using summarized data.

*Genetic Epidemiology*

**Comment 4**

Would be interesting to see if restricting the random pair analysis to certain covariates reduces the shrinkage. For example, what if the random pairs are taken from the same geographical region or the same city (but not the same coordinates). At one extreme, it would be interesting to construct random pairs that are from the closest coordinates (but not the same).

The reviewer has suggested comparing spouses to “random” individuals matched on covariates. The purpose of the random pair analysis is to generate an empirical distribution of correlations that we could compare to the observed spousal distribution. It is possible to also conduct stratified random matching to provide information about the covariates that explain the shrinkage. However, this would require substantial additional analysis and is outside the scope of this paper.

**Comment 5**

The authors compare the shrinkage in PRS, but what about the effect on individual SNPs? Do they have enough power to look at the heterogeneity in shrinkage across the different SNPs?

As the reviewer noted, we reported the average shrinkage across many genetic variants associated with phenotypes of interest rather than the shrinkage for individual variants.

However, for alcohol, we used a single genetic variant in ADH1B, which is known to have a large effect on alcohol consumption. Here, the WSP shrinkage estimate was 14% (95% C.I. -5%, 33%) with a standard error of around 10%. This imprecise shrinkage estimate suggests that there will be limited power to test for heterogeneity in shrinkage of individual SNPs (which will have far smaller effects than this example).

A further complication with testing on the individual variant level is the potential issue of weak genetic associations, i.e. if a genetic variant has a beta value very close to zero in the random-pair model. This is more likely to occur with low statistical power and would be problematic for estimating shrinkage because shrinkage is a function of a ratio. For example, depending on the beta value in the WSP model, the shrinkage point estimate for that variant could be a large negative number with a very wide confidence interval, distorting any estimates of heterogeneity.

Shrinkage is $1-\frac{WSP estimate}{Random pair estimate}$ so as the random-pair estimate tends to zero, then the ratio will tend to infinity if the WSP estimate > 0.

The additive approach of combining all the variants into a polygenic score, circumvents this issue but as the reviewer noted, has the limitation of not being informative about SNP-level heterogeneity.

**Reviewer 2**

 This study presents a comparison of two different “within-family” models for testing genetic associations. The concept is clear that a within-spouse pair analysis may account for shared environment as the shared environment among spouses is the same. However, I have a few concerns:

**Comment 1**

I do not see how a WSP model controls for ancestry confounding if spousal differences correlate with their ancestry differences? Yes, pairs may be matched for the home environment, but a correlation between delta_phenotype and delta_genotype could certainly be driven by delta_ancestry among spousal pairs, perhaps even more so as all other environmental similarity may be controlled for. If one fits the model in a mixed model framework does it help control for stratification? What if there is a phenotypic correlation with PC1 and mating is random with respect to PC1, is there covariance between the effect size estimates and the PC loadings. The sibling pair analysis, due to mendelian segregation should be unbiased of this, but I do not see a strong consideration of genetic stratification effects in this work, which I feel leaves it lacking.

Within-sibship models will perfectly control for ancestral differences because both siblings will be drawn from the same distributions of parental ancestry. In contrast, spouses are unlikely to be drawn from identical ancestries. However, spouses often assort on geography and other factors relating to ancestry so are likely to be more ancestrally similar than two random individuals from the population. For example, this was illustrated by previous work referenced in the manuscript (Testing for non-random mating: evidence for ancestry-related assortative mating in the Framingham heart study. Genet Epidemiol. 2010;34(7):674-9.) as providing evidence for the ancestral similarities of spouses.

Considering that the within-sibship model, where two siblings have the same parents and ancestry completely removes population stratification bias, it follows that analyes comparing individuals with similar ancestry (e.g. WSP or a region stratified GWAS) will partially remove population stratification bias because if some individuals have the same value for the confounder, then this cannot induce bias. Indeed, we investigated this in Model A in the simulations, showing that bias from a confounder in the WSP model tends to zero as spousal correlations for the confounder tend to one.

To investigate further the ancestral similarities of spouses, we have reported the within-pair (spouse, sibling) associations for the first 10 Principal Components in Supplementary Table 4.

**Supplementary Table 4** Spouse and sibling pair correlations for principal components

| **Principal component** | **Spousal pair correlation (95% C.I.)** | **Sibling pair correlation**  **(95% C.I.)** |
| --- | --- | --- |
| PC1 | 0.16 (0.15, 0.17) | 0.94 (0.94, 0.94) |
| PC2 | 0.11 (0.10, 0.12) | 0.87 (0.87, 0.88) |
| PC3 | 0.26 (0.25, 0.27) | 0.90 (0.90, 0.91) |
| PC4 | 0.32 (0.31, 0.33) | 0.98 (0.97, 0.98) |
| PC5 | 0.32 (0.31, 0.33) | 0.97 (0.97, 0.98) |
| PC6 | 0.10 (0.10, 0.11) | 0.76 (0.75, 0.77) |
| PC7 | 0.22 (0.20, 0.23) | 0.90 (0.90, 0.91) |
| PC8 | 0.20 (0.19, 0.21) | 0.89 (0.89, 0.90) |
| PC9 | 0.30 (0.29, 0.31) | 0.91 (0.91, 0.92) |
| PC10 | 0.17 (0.16, 0.18) | 0.74 (0.73, 0.75) |

We added the following to the text in the results section:

“We first explored the extent to which spouse and sibling pairs are correlated for the first 10 principal components to inform the extent of pairwise spousal ancestral similarities. Spouse pairs were correlated for these first 10 principal components with correlations ranging from 0.10 for PC6 to 0.32 for PC4. As expected, sibling pairs were very strongly correlated for the first 10 principal components with correlations ranging from 0.74 for PC10 to 0.98 for PC4 (**Supplementary Table 4**).”

These correlations indicate that the ancestral similarities of spouses are likely to partially control for population structure, as sibling models do. We are not claiming that within spousal analyses will perfectly control for ancestral, population or familial differences as effectively as siblings. We are arguing that within spousal analyses are likely to provide better control of familial and demographic differences than studies using unrelated individuals and therefore are a potentially useful model when triangulating evidence across different study designs.

**Comment 2**

Why use summary statistics from previously published non-UKB meta-analyses? Why not compare to directly estimating the effects using a mixed-effects association model in the UK Biobank? Non-UKB meta-analysis estimates are obtained from very heterogeneous cohorts in the worst type of association study methodology (single-marker marginal associations with some arbitrarily determined number of PCs). The current comparison is valid, but I think a comparison of analysing the same data in a different way is also warranted. Note systolic blood pressure was analysed in this way (within UKB analysis) and the shrinkage, especially from the spouse pair model is much less than for some of the other traits.

We used summary statistics from external GWAS because these are some of the largest studies available and so have more precise association estimates than GWAS from UK Biobank alone. Using external GWAS also largely eliminates the possibility of bias from sample overlap.

The SNPs from the discovery sample we used were all identified at the conservative genome wide significance threshold, so the vast majority are likely to be genuine associations rather than false positives. The lack of shrinkage of systolic blood pressure is highly consistent with our results from a recent within-sibship GWAS (LJ Howe et al. 2021 biorXiv), and other studies.

Furthermore, the method used to derive shrinkage involves comparing the polygenic score association in the random-pair sample with the same association in the WSP or within-sibship model. Therefore, the weights from the discovery GWAs will be in both the numerator and denominator and so issues in the discovery GWAS should not induce bias in shrinkage estimate (which is a function of the ratio).

**Comment 3**

I would also suggest rather than taking the most statistically significant estimates, which ascertains a very specific subset of variants with specific LD and MAF properties, that comparisons are done across a range of markers. Perhaps prune the data for LD beforehand and analyse all the markers and then observe the shrinkage genome-wide and as a function of the association test statistic values?

We decided to focus on WSP and within-sibship shrinkage for the variants most strongly associated with each phenotype for several reasons.

First, the method we used to estimate shrinkage involved comparing polygenic score associations with the scores weighted by their effect sizes from the discovery GWAS. For many variants, the weight will be close to zero and so they will not greatly impact the analysis if they are included. Following on from this point, conceptually estimating shrinkage requires there to be something to shrink. If a genetic variant has a very null association with the phenotype in the random-pair model, can the true association really shrink from basically zero. In practice, shrinkage is estimated as a function of the ratio so if the numerator is close to zero, the shrinkage estimates will tend to infinity.

Shrinkage is $1-\frac{WSP estimate}{Random pair estimate}$ so as the random-pair estimate tends to zero, the ratio will tend to infinity if the WSP estimate > 0.

Third, our intention was to use the shrinkage estimates to make inferences about each phenotype. The genome-wide significant variants are likely to be the most relevant to the aetiology of that phenotype. As more and more variants are included, the interpretation becomes more complex. This is because the mechanistic pathways underlying the genetic associations are likely to be more distal and pleiotropic. Given that we’re interested in mechanisms acting on phenotypes such as assortative mating, it seems sensible to use variants that are known to be strongly associated with phenotypes rather than every variant in the genome, which may or may not be associated with the phenotype.

We suspect that the reviewer is interested in whether there are systematic differences in genetic associations across the genome, rather than simply at the strongest association signals. This is a distinct question to shrinkage (i.e., reduction in association), because it is more agnostic about the direction of difference (i.e., do associations become stronger or weaker). We have thought long and hard about the best way of estimating such differences, and it is non-trivial to estimate this systematically across the genome. The main issue is that the individual genetic association estimates have large standard errors and so there’s a lot of variance in association estimates between the models based on chance alone. In the shrinkage analysis, we can combine the average shrinkage across many variants to get the average shrinkage. Here, chance will lead to differences in either direction so will not bias shrinkage. However, when looking at simply differences in either direction, the average absolute difference between association estimates greatly depends on the sample size and statistical power of the dataset.

**Reviewer 3**

In the manuscript Howe et al., titled “Assortative mating and within-spouse pair comparisons”, the authors demonstrated the potential of collider bias in within-spouse pair (WSP) study design, such that in GWAS estimating effect sizes using WSP design there is a greater degree of shrinkage compared to the gold standard within-sibship design. The paper is relatively lightweight, consisting of two or three analyses total, but I think the paper potentially brings awareness of an important topic in genetic study design. On the other hand though, despite the application in GWAS, the language of the paper seems to be tailored more towards an epidemiology audience, and might not be as familiar to human genetics community (i.e. this reviewer).

**Comment 1**

While I am not a domain expert on causal inference, I can appreciate the investigation into sources of bias in a GWAS. However, it is always easier for me (and I assume for the larger readership) to think about confounding bias, rather than collider bias. Thus the first set of comments I have are related to presentation.

**Comment 1a**

In the Introduction it would be helpful to be more explicit about how WSP design is used in genetic studies (this is submitted to a Genetics journal after all). Currently it only became more apparent how WSP (and within-sibship) designs are modeled later in the paper with the empirical example and would require the reader to go into an even later Methods section to understand the details.

We have rewritten the introduction to shift the focus from solely spouses and assortative mating to start by discussing within-sibship genetic association models (a design many in genetics will be familiar with) and then following on with how WSP models may have similar advantageous properties. Please see the before and after below.

Before:

“Human spouses are often similar for many traits [1]. One mechanism underlying spousal similarities is that cohabitating spouses share a common environment during their relationship [2] which may act to increase phenotypic similarity, such as for behavioural (e.g., physical activity and alcohol use) or personality traits [3, 4]. The shared adulthood environment between spouses has prompted the use of spouses in a variety of contexts in epidemiological and genetic research using a model that we refer to as the “within-spouse pair” (WSP) model. The WSP model involves modelling the similarities and differences of spouses, either by analysing the differences between each pair or by modelling spousal relationships as a covariate in a fixed-effect model. For example, previous studies have used the WSP model to estimate effects of the shared adulthood environment [5-11]. The WSP model has also been proposed as an approach to reduce confounding in aetiological research, with environmental confounders likely to be strongly correlated between spouses [12].”

After:

“Within-sibship models have been widely used in genetic association studies for many decades [13-18]. Genotypic differences between siblings are the consequence of random segregation at meiosis, rather than parental or ancestral differences, and so within-sibship genetic association models control for demography (assortative mating, population stratification) and indirect genetic effects of parents [16, 17, 19]. However, there is a paucity of genetic data from siblings with limited availability for many phenotypes. Furthermore, while siblings are matched on the early-life environment, their environments in adulthood may differ.

In contrast, spouses may have different early-life environments but are likely to share an environment for much of adulthood while cohabiting [2]. This may act to increase phenotypic similarity, such as for behavioural (e.g., physical activity and alcohol use) or personality traits [3, 4]. The shared adulthood environment between spouses has prompted their use in a variety of contexts in genetic and epidemiological research using a model that we refer to as the “within-spouse pair” (WSP) model. The WSP model involves modelling the similarities and differences of spouses, either by analysing the differences between each pair or by modelling spousal relationships as a covariate in a fixed-effect model. For example, previous studies have used the WSP model to estimate phenotypic variance explained by the shared adulthood environment [5-11]. The WSP model has also been proposed as an approach to reduce confounding in aetiological research, with environmental confounders likely to be strongly correlated between spouses [12]. Here, we describe the strengths and limitations of within-spouse (WSP) designs for genetic studies.”

**Comment 1b**

It would be better to have an explicit (and more intuitive) real world example of collider bias that will help the reader appreciate the bias. The current example “association between factors which induce disease incidence are likely to be distorted in samples of only disease cases” provided little context. The spousal compatibility example is an improvement, but already assumed some level of understanding of collider bias to make sense.

We agree with the reviewer that the introduction would have benefitted from more intuitive examples of collider bias. We have therefore removed the disease cases example and added in an example on school scholarships and an example involving spousal similarities for age and education. Please see below.

Before:

“Collider bias occurs when conditioning on a variable which is influenced by two or more factors. This bias can induce spurious associations between these factors following such conditioning analysis, or in the selected sample. For example, associations between factors which induce disease incidence are likely to be distorted in samples of only diseased cases [20, 21]. Similarly, spousal samples condition on spousal compatibility, a pairwise measure of how likely two individuals are to enter a relationship. If spousal compatibility is a function of phenotypic similarity across multiple phenotypes, then collider bias could potentially arise in the WSP model (**Figure 1**) [20, 22, 23].”

After:

“The WSP model may be susceptible to collider bias, which can occur when conditioning on a variable which is influenced by two or more upstream factors. Collider bias can induce spurious associations between these factors where the collider variable is conditioned on in analysis either by analytical model design or sample selection. For example, if a school grants scholarships to either individuals who are exceptional at sport or exceptional academically, then sporting and academic ability will be negatively correlated amongst individuals with scholarships. Similarly, spousal samples condition on spousal compatibility, a pairwise measure of how likely two individuals are to enter a relationship. If several phenotypes influence spousal compatibility, then collider bias could potentially arise in the WSP model [20, 22, 23]. For example, if similarities for age and educational attainment influence compatibility then spouses with larger age differences are more likely to have similar educational attainment (**Figure 1**).”

**Comment 1c**

Similarly, Figure 1 can use some real world context. In confounding, chopsticks use or human height are commonly used example for potential confounding. It would be helpful to have something like that to illustrate collider bias.

We have changed Figure 1 to use the example from the text of age and education differences between spouses. Hopefully, this better illustrates the potential associations induced by conditioning on assorted spouses. For example, if spouses have a large age difference, they must be compatible in some other way such as having a similar education.

Updated Figure 1:

**Comment 1d**

Generally, the writing can be more concrete at times. For example, “the two exposures could be correlated in the WSP model dependent on underlying model and the degree of assortment on the exposure” is a true statement, but really covered all possibilities in one sentence. Again a real world intuitive example would be helpful.

We have edited throughout for conciseness and clarity and re this specific sentence have edited to read as follows, including a real world example to illustrate the point:

“Since there is assortment on the two exposures, assortment acts as a collider, which induces associations between variables that would otherwise be independent in the population. For example, in the WSP model, assortment on height and educational attainment could induce a negative correlation between height and educational attainment genetic scores.”

**Comment 1e**

I also think the direction of bias is important to be explicit when introducing collider bias with examples. To draw parallel from confounder, I can state that genetic ancestry is correlated with both the genotype (in frequency cline) and phenotype (differences in height across latitude), which results in confounding. But it is much more illustrative to say that because height value is larger in Northern Europe than in Southern Europe, alleles that are higher frequency in Northern Europe than Southern Europe will appear to be associated with increasing height if the confounder (genetic ancestry) is not adjusted. Can an example be given for collider bias, which would also provide the intuition of why there should be a shrinkage in estimates later.

We were initially being cautious about the direction of bias because it does depend very heavily on the underlying true causal model which is difficult to evaluate. However, we have tweaked the discussion to better illustrate why we think that collider bias would lead to shrinkage in genetic associations.

Before:

“Collider bias induced by comparing assorted pairs could contribute to the observed shrinkage depending on the interactive model between the colliding effects and the degree of assortment. Assuming additive non-interactive effects, collider bias is likely to shrink rather than inflate genetic associations because assortment would induce negative correlations in the WSP model between determinants of the assorted trait, such as between genetic variants.”

After:

“Collider bias induced by comparing assorted pairs could contribute to the observed shrinkage depending on the interactive model between the colliding effects and the degree of assortment. Assuming additive non-interactive effects, collider bias is likely to shrink rather than inflate genetic associations because assortment would induce negative correlations in the WSP model between factors influencing the assorted trait in the same direction. For example, assortment could induce negative correlations between height increasing genetic variants and height increasing environmental factors in the WSP model, leading to shrinkage when estimating WSP genetic associations.”

**Comment 2**

“This suggests that population stratification is unlikely to explain the shrinkage in these estimates.” I failed to see the logic of this sentence, as it is referring to the alcohol analysis with a single, stratified, SNP. Perhaps the authors meant for this sentence to follow the previous sentence (that including PCs changed the results only a little).

We have reordered this section to read:

“This suggests that population stratification is unlikely to explain the shrinkage in these estimates (**Table 1**). However, note that the alcohol analysis included only a single SNP which is known to be strongly associated with population structure in UK Biobank [24].”

**Comment 3**

It is interesting to note that for height, shrinkage under WSP model (even without PC) is only slightly bigger than shrinkage in within-sibship estimates. The authors attributed the additional shrinkage due to collider bias. I treat the shrinkage from within-sibship design as entirely driven by confounding due to population structure. But WSP is not otherwise modeling population structure, so the fact that the shrinkage is quite close implies that collider bias (at least for height) due to assortative mating is relatively minor, and most of the shrinkage is driven by population structure that WSP also controls?

Population structure seems unlikely to explain all of the within-sibship/WSP shrinkage we observe for height – the estimates without controlling for principal components are statistically indistinguishable from the estimates controlling for the PCs. The observed within-sibship shrinkage is likely to also be explained by assortative mating and indirect genetic effects of parents (especially for educational attainment). We interpret the WSP shrinkage as likely a consequence of both assortative mating controlling for environmental factors and collider bias.

As discussed in our response to comment 5 of reviewer 3, we have now included external within-sibship shrinkage estimates from a consortium-based within-sibship GWAS, which are more precise than the estimates previously presented in our study. Using these estimates there is clearer evidence that the WSP shrinkage estimates for height, BMI and education are larger than the within-sibship estimates, likely indicating collider bias.

**Comment 4**

On a similar line of reasoning, without explicitly modeling population structure in WSP, the fact that it appears to capture so much of the impact due to confounding from population structure is it because the analysis was done in a relatively homogeneous European subset of UKB? What would happen if the authors repeated the analysis using all of the UKB, including the non-Europeans, such that the impact due to structure should be much stronger? Would shrinkage from WSP now be smaller than shrinkage from within-sibship (because WSP is failing to control for structure completely)?

This is an interesting suggestion from the reviewer and would be certainly of theoretical interest for understanding the extent to which the WSP model accounts for population structure.

However, there are a few major practical issues that would make interpreting the analysis challenging. First, the proportion of UK Biobank with non-European ancestry is relatively modest so the shrinkage estimates may not change too much even if the WSP model was partially controlling for ancestry. Second, the genetic variants for each phenotype were identified in European populations and so may not be strongly associated with these phenotypes in non-Europeans because of linkage disequilibrium differences.

These issues mean that including individuals of non-European ancestry in shrinkage analyses, would require substantial thought, beyond the scope of this manuscript.

As discussed in our response to the first comment of reviewer 2, we have now included estimates of both spousal and sibling principal component correlations which indicate that the WSP model may partially control for population structure.

Please see Supplementary Table 4 below which shows strong evidence of correlations between spouses for principal components but that the correlations are much smaller than for siblings.

**Supplementary Table 4** Spouse and sibling pair correlations for principal components

| **Principal component** | **Spousal pair correlation (95% C.I.)** | **Sibling pair correlation**  **(95% C.I.)** |
| --- | --- | --- |
| PC1 | 0.16 (0.15, 0.17) | 0.94 (0.94, 0.94) |
| PC2 | 0.11 (0.10, 0.12) | 0.87 (0.87, 0.88) |
| PC3 | 0.26 (0.25, 0.27) | 0.90 (0.90, 0.91) |
| PC4 | 0.32 (0.31, 0.33) | 0.98 (0.97, 0.98) |
| PC5 | 0.32 (0.31, 0.33) | 0.97 (0.97, 0.98) |
| PC6 | 0.10 (0.10, 0.11) | 0.76 (0.75, 0.77) |
| PC7 | 0.22 (0.20, 0.23) | 0.90 (0.90, 0.91) |
| PC8 | 0.20 (0.19, 0.21) | 0.89 (0.89, 0.90) |
| PC9 | 0.30 (0.29, 0.31) | 0.91 (0.91, 0.92) |
| PC10 | 0.17 (0.16, 0.18) | 0.74 (0.73, 0.75) |

**Comment 5**

Discussion, paragraph 2: the authors are attributing additional shrinkage to collider. I think it should be stressed here that there is no statistically detected evidence of heterogeneity between WSP and within-sibship analysis. So it is possible that there is no collider bias in real world example. Can the authors demonstrate the existence of collider bias in real world genetic data?

We agree with the reviewer that in the previously presented results, there was limited evidence for heterogeneity between the WSP and within-sibship shrinkage estimates.

However, as discussed in the response to the first reviewer, we have now added within-sibship shrinkage estimates from a recent Within-sibship GWAS preprint (Within-sibship GWAS provide improved estimates of direct genetic effects LJ Howe et al 2021, biorXiv) to the manuscript in Supplementary Table 5. This study estimated shrinkage using similar methods but in a much larger consortium sample and so the within-sibship shrinkage estimates are much more precise. Please see Supplementary Table 5 below.

**Supplementary Table 5** Comparisons of WSP and within-sibship shrinkage estimates.

| **Phenotype** | **Shrinkage: % (95% C.I.)** | | |
| --- | --- | --- | --- |
|  | **WSP**  **(Including PC1-10)** | **Within-sibship**  **(Including PC1-10)** | **Within-sibship ***  **(Sibling GWAS)** |
| Height | 17% (14%, 20%) | 13% (8%, 18%) | 10% (8%, 12%) |
| Educational attainment | 71% (62%, 79%) | 51% (33%, 70%) | 46% (40%, 52%) |
| BMI | 16% (6%, 25%) | 5% (-12%, 22%) | 0% (-5%, 5%) |
| SBP | 0% (-8%, 7%) | 5% (-7%, 18%) | 0% (-6%, 6%) |
| Alcohol consumption | 14% (-5%, 33%) | 4% (-46%, 54%) | -3% (-28%, 22%) |

* Note that these estimates are taken from a biorXiv preprint (*Within-sibship GWAS provide improved estimates of direct genetic effects*. LJ Howe et al 2021). The shrinkage estimates are from the polygenic score analysis including genetic variants with P < 1x10^-5^.

The more precise (consortium) within-sibship shrinkage estimates are both consistent with our previous (UK Biobank only) within-sibship shrinkage estimates and (based on non-overlapping confidence intervals) clearly smaller than the WSP shrinkage estimates for height, BMI and educational attainment. The within-sibship model will completely control for population stratification, assortative mating and indirect genetic effects of parents, so the WSP shrinkage estimates being higher than the within-sibship estimates seems most likely to be a consequence of collider bias.

**Comment 6**

It seems that collider bias, as investigated here due to assortative mating, is only the result when conditioning on the assortment (i.e. specifically in a WSP design). Biobank data often contains incidental spousal relationships that are not actively investigated or filtered. Can the authors discuss the impact of this more explicitly? If collider bias is only a concern in WSP designs, what implications are there in the standard GWAS design? The authors appeared wanting to discuss this on Page 12, but were quite terse.

Yes, we believe that the specific form of collider bias relating to assortative mating will only be induced if comparing assorted spouses in the WSP model. We have edited the discussion of the manuscript to clarify that the non-randomness of spouses could induce selection bias.

Before: A further implication is that assortative mating is likely to contribute to the phenotypic and genetic structure of epidemiological studies. Large studies such as the UK Biobank, frequently incidentally sample participants who are partnered with another study participant [24]. These individuals will be non-independent. The consequences of jointly enrolled spouses are unclear but are one mechanism influencing study participation.

After: A further implication is that assortative mating is likely to contribute to the phenotypic and genetic structure of epidemiological studies. Large studies such as the UK Biobank, frequently incidentally sample participants who are partnered with another study participant [24]. The non-randomness of study participation in UK Biobank has been previously discussed as a possible cause of selection (participation) bias. Our findings illustrate that assortative mating is likely to contribute to the non-random distribution of phenotypes (and genotypes) in population biobanks.

**Comment 7**

Table S3 is rather uninformative. For example, mean height is given without regards to sex, which would clearly impact the mean. The table also does not provide any intuitive sense of assortment between spouses. It would be better to provide scatterplots of the phenotypes for each pair of spouse, and provide mean and sd at least by sex.

Thanks for this comment, we agree with the reviewer. We have expanded Table S3 to present sex-stratified means and standard deviations. We have also generated scatterplots of the continuous phenotypes between sexes as Supplementary Figures.

**References**

1.	Buss DM. Human mate selection: Opposites are sometimes said to attract, but in fact we are likely to marry someone who is similar to us in almost every variable. American Scientist. 1985;73(1):47-51.

2.	Davey Smith G. Epidemiology, epigenetics and the ‘Gloomy Prospect’: embracing randomness in population health research and practice. International Journal of Epidemiology. 2011;40(3):537-62.

3.	Ask H, Rognmo K, Torvik FA, Roysamb E, Tambs K. Non-random mating and convergence over time for alcohol consumption, smoking, and exercise: the Nord-Trondelag Health Study. Behav Genet. 2012;42(3):354-65. Epub 2011/10/19. doi: 10.1007/s10519-011-9509-7. PubMed PMID: 22005768.

4.	Ask H, Idstad M, Engdahl B, Tambs K. Non-random Mating and Convergence Over Time for Mental Health, Life Satisfaction, and Personality: The Nord-Trøndelag Health Study. Behavior Genetics. 2013;43(2):108-19. doi: 10.1007/s10519-012-9578-2.

5.	Xia C, Amador C, Huffman J, Trochet H, Campbell A, Porteous D, et al. Pedigree-and SNP-associated genetics and recent environment are the major contributors to anthropometric and cardiometabolic trait variation. PLoS Genetics. 2016;12(2):e1005804.

6.	Hill WD, Arslan RC, Xia C, Luciano M, Amador C, Navarro P, et al. Genomic analysis of family data reveals additional genetic effects on intelligence and personality. Molecular Psychiatry. 2018;23(12):2347.

7.	Amador C, Xia C, Nagy R, Campbell A, Porteous D, Smith BH, et al. Regional variation in health is predominantly driven by lifestyle rather than genetics. Nature Communications. 2017;8(1):801.

8.	Wang K, Gaitsch H, Poon H, Cox NJ, Rzhetsky A. Classification of common human diseases derived from shared genetic and environmental determinants. Nature Genetics. 2017;49(9):1319.

9.	Barbu MC, Shen X, Walker RM, Howard DM, Evans KL, Whalley HC, et al. Epigenetic prediction of major depressive disorder. Molecular Psychiatry. 2020. doi: 10.1038/s41380-020-0808-3.

10.	Zeng Y, Amador C, Xia C, Marioni R, Sproul D, Walker RM, et al. Parent of origin genetic effects on methylation in humans are common and influence complex trait variation. Nature Communications. 2019;10(1):1383. doi: 10.1038/s41467-019-09301-y.

11.	Zeng Y, Navarro P, Xia C, Amador C, Fernandez-Pujals AM, Thomson PA, et al. Shared Genetics and Couple-Associated Environment Are Major Contributors to the Risk of Both Clinical and Self-Declared Depression. EBioMedicine. 2016;14:161-7. Epub 2016/11/14. doi: 10.1016/j.ebiom.2016.11.003. PubMed PMID: 27838479; PubMed Central PMCID: PMCPMC5161419.

12.	Bjorngaard JH, Vie GA, Krokstad S, Janszky I, Romundstad PR, Vatten LJ. Cardiovascular mortality - Comparing risk factor associations within couples and in the total population - The HUNT Study. International journal of cardiology. 2017;232:127-33. Epub 2017/01/14. doi: 10.1016/j.ijcard.2017.01.041. PubMed PMID: 28082089.

13.	Curtis D, Miller MB, Sham PC. Combining the sibling disequilibrium test and transmission/disequilibrium test for multiallelic markers. American journal of human genetics. 1999;64(6):1785.

14.	Abecasis GR, Cardon LR, Cookson WO. A general test of association for quantitative traits in nuclear families. Am J Hum Genet. 2000;66(1):279-92. Epub 2000/01/13. doi: 10.1086/302698. PubMed PMID: 10631157; PubMed Central PMCID: PMCPMC1288332.

15.	Fulker DW, Cherny SS, Sham PC, Hewitt JK. Combined linkage and association sib-pair analysis for quantitative traits. Am J Hum Genet. 1999;64(1):259-67. Epub 1999/01/23. doi: 10.1086/302193. PubMed PMID: 9915965; PubMed Central PMCID: PMCPMC1377724.

16.	Lee JJ, Wedow R, Okbay A, Kong E, Maghzian O, Zacher M, et al. Gene discovery and polygenic prediction from a genome-wide association study of educational attainment in 1.1 million individuals. Nat Genet. 2018;50(8):1112-21. Epub 2018/07/25. doi: 10.1038/s41588-018-0147-3. PubMed PMID: 30038396; PubMed Central PMCID: PMCPMC6393768.

17.	Howe LJ, Nivard MG, Morris TT, Hansen AF, Rasheed H, Cho Y, et al. Within-sibship GWAS improve estimates of direct genetic effects. bioRxiv. 2021:2021.03.05.433935. doi: 10.1101/2021.03.05.433935.

18.	Brumpton B, Sanderson E, Hartwig FP, Harrison S, Vie GÅ, Cho Y, et al. Within-family studies for Mendelian randomization: avoiding dynastic, assortative mating, and population stratification biases. Nature Communications. 2020:602516.

19.	Young AI, Benonisdottir S, Przeworski M, Kong A. Deconstructing the sources of genotype-phenotype associations in humans. Science. 2019;365(6460):1396-400. doi: 10.1126/science.aax3710 %J Science.

20.	Munafò MR, Tilling K, Taylor AE, Evans DM, Davey Smith G. Collider scope: when selection bias can substantially influence observed associations. International Journal of Epidemiology. 2017.

21.	Paternoster L, Tilling K, Davey Smith G. Genetic epidemiology and Mendelian randomization for informing disease therapeutics: Conceptual and methodological challenges. PLoS Genet. 2017;13(10):e1006944. Epub 2017/10/06. doi: 10.1371/journal.pgen.1006944. PubMed PMID: 28981501; PubMed Central PMCID: PMCPMC5628782.

22.	Catalogue of bias collaboration, Lee H, Aronson JK, D N. Collider bias. In Catalogue of Bias. 2019. Available from: https://catalogofbias.org/biases/collider-bias/.

23.	Lawlor DA, Tilling K, Davey Smith G. Triangulation in aetiological epidemiology. International Journal of Epidemiology. 2016;45(6):1866-86.

24.	Howe LJ, Lawson DJ, Davies NM, Pourcain BS, Lewis SJ, Davey Smith G, et al. Genetic evidence for assortative mating on alcohol consumption in the UK Biobank. Nature Communications. 2019. doi: 10.1038/s41467-019-12424-x.
