# Supplementary material for: Assortative mating and within-spouse pair comparisons
Source: PLoS Genet. 2021 Nov 4;17(11):e1009883. doi: 10.1371/journal.pgen.1009883 (PMC8594845; doi:10.1371/journal.pgen.1009883)
Supplement: Attachment — Submitted filename: WithinSpouse_Response_September2021.docx [file pgen.1009883.s013.docx]

Laurence J Howe

Laurence.Howe@bristol.ac.uk

MRC Integrative Epidemiology Unit

University of Bristol

Bristol, BS8 2BN

29^th^ September 2021

David Balding

Methods Editor

*PLOS Genetics*

Dear Professor Balding,

I’m writing regarding the manuscript “*Assortative mating and within-spouse pair comparisons*”. We would like to thank the journal, editors and reviewers for their positive feedback on the manuscript and the opportunity to revise and resubmit.

We have responded below to the editor and reviewer comments, detailing changes made to the manuscript.

We hope that the revised manuscript is suitable for publication in *PLOS Genetics*. Thank you for considering our manuscript.

Yours sincerely,

Dr Laurence Howe on behalf of the authors

**Editor comments:**

The manuscript was fully evaluated at the editorial level and by independent peer reviewers.  Both reviewers find that the revised article has improved considerably and has substantial merit, but Reviewer 3 raises a fundamental question about the role of collider bias in the WSP design and how it impacts the control of confounding in an empirical setting.  The editors would like to see these addressed. The reviewer suggest two possible ways to quantify the problem. Either (or both) of these additional analyses would potentially strengthen the paper and help drive home its main argument.  Alternatively the authors may find a better approach to address Reviewer 3's concerns.

We thank the editor and reviewers for their useful feedback on the manuscript. We have responded to the individual reviewer comments below.

**Reviewer #2:**

I believe that the authors have argued for the approach taken in this manuscript very well and in doing so have alleviated my concerns sufficiently for meta support publication. While I think more work needs to be done on this topic, what is presented here is very interesting and represents an ideal starting point for future research.

We thank the reviewer for their helpful and supportive feedback which has helped to greatly improve the manuscript.

**Reviewer #3:**

I appreciate the authors significantly reworked the presentation of the manuscript, which in my view is now much easier to follow. I think both the Introduction and Discussion sections are now much improved.

We thank the reviewer for their helpful and supportive feedback which has helped to greatly improve the manuscript.

My main concern is the following. The manuscript strives to drive home two points (based on the abstract): (1) WSP design can reduce confounding, but (2) WSP design is susceptible to collider bias. Both confounding and collider bias are addressed in simulation, but there is no direct evidence of collider bias is at play in empirical data analysis. Rather, the authors inferred that collider bias is happening because shrinkage of the effect size estimates in WSP design is larger than within-sibship design; since within-sibship design is perfectly controlling for confounding due to ancestry/demography/family, the additional shrinkage is attributed to collider bias that induces a negative correlation. But ultimately, this is still only an indirect inference by deduction. Collider bias is thus, in my view, still not strongly supported by empirical data analysis. Would some approach like that published in Day et al., AJHG 2016 (PMID 26849114) be feasible in this setting to provide a direct demonstration of collider bias?

As the reviewer articulated, the evidence for collider bias affecting the WSP model is based on simulated data and indirect inference from the difference between WSP and within-sibship estimates rather than direct evidence. Demonstrating collider bias in empirical data is challenging as there are often many potential mechanisms that could affect estimates.

The example by Day et al interestingly uses associations between autosomal genetic variants and sex to illustrate collider bias, because autosomal variants cannot plausibly influence sex so any associations must relate to collider bias.

We used a similar approach to evaluate collider bias in the WSP model by comparing the associations of age with blood pressure and coronary artery disease risk between the within-spouse and within-random pair models. Age, like sex, cannot be modified by autosomal genetic variants or phenotypes, so associations between age and an outcome can only be plausibly distorted by selection/collider bias. Finding no differences between the age estimates, we concluded little evidence of collider bias in this instance in the discussion on page 11. Although we find minimal evidence of collider bias in this specific age analysis, this doesn’t rule out collider bias in other analyses such as the genetic association analysis.

“However, in the negative control example of age on health outcomes, we found little evidence of collider bias; within-pair effect estimates of age on CAD and SBP were consistent between spouse and non-spouse pair samples.”

Although our inference that collider bias may be downwardly biasing the WSP genetic association estimates is indirect, we argue that the evidence is still relatively strong. The WSP genetic association estimates shrinking by a greater magnitude than the within-sibship estimates cannot reflect a “true” shrinkage (i.e. reduction in confounding), because the within-sibship model controls for confounding much more effectively than the WSP model. The difference in shrinkages between the two models may reflect some form of collider/selection bias. In the manuscript we also discussed how assortative mating has been shown to have induced correlations between spousal phenotypes, and between height increasing alleles on odd and even chromosomes in the same individual. It follows that assortative mating may induce correlations between spousal differences for phenotypes and genotypes. Such correlations could then induce some degree of collider bias in the WSP model.

On page 11: “For example, assortment could induce negative correlations between height increasing genetic variants and height increasing environmental factors in the WSP model, leading to shrinkage when estimating WSP genetic associations. This is in contrast to the population-level effects of assortative mating which inflate associations because of induced positive correlations between trait-increasing variants on different chromosomes [22].”

We have added the following to the manuscript to clarify that the age analysis is a similar premise to the autosomal GWAS of sex example that the reviewer mentioned.

Page 9 – Age on SBP/CAD negative control analysis

“This is a similar premise to autosomal GWAS of sex, where genetic associations are likely to reflect participation bias because autosomal genetic variation cannot influence sex [38].”

We have also added a sentence to the discussion to improve our argument regarding the indirect evidence for collider bias in the WSP model.

Before: The WSP shrinkage estimates being higher than the sibling estimates suggests that the shrinkage cannot be explained by adjustment for confounding alone. If the only source of shrinkage is adjustment for confounding, then WSP shrinkage estimates should be smaller than within-sibship estimates because spouse models are unlikely to fully control for demographic or family-level (e.g. parental nurture) effects.

After: The WSP shrinkage estimates being higher than the sibling estimates suggests that the shrinkage cannot be explained by adjustment for confounding alone. If the only source of shrinkage is adjustment for confounding, then WSP shrinkage estimates should be smaller than within-sibship estimates because spouse models are unlikely to fully control for demographic or family-level (e.g. parental nurture) effects. **Collider bias induced by comparing assorted pairs is one potential explanation for the observed WSP shrinkage.**

In fact, by the author’s argument, shrinkage in the WSP design need not be greater than that in the within-sibship design for collider bias to be at play, since the correlation between spouses in principal component space is quite a bit less than the correlation between sibs, suggesting that WSP design is much less efficacious to control for confounding (at least due to ancestry), than the within-sibship design. Yet, it is not clear the degree to which WSP design could be controlling for confounding, while additional shrinkage could be attributed to collider bias. So some form of partitioning of the effects attributed to correction for confounding could contribute to evidence that collider bias is operating (though still indirectly).

We agree that it would be useful to be able to partition the WSP shrinkage into confounding reduction and collider bias. However, the main obstacle to estimating these parameters in empirical data is the challenge in quantifying the extent to which spouses are correlated for confounders. We attempted to provide some insight into this by evaluating spousal correlations for genomic principal components and have in response to the reviewer’s comments also computed spousal correlations for birth coordinates. These correlations are informative about how the WSP model may account for population stratification. However, it is difficult to quantify spousal correlations for other potential confounders such as indirect genetic effects of parents. Parental phenotypes such as socioeconomic position are often correlated between spouses but suitable data to quantify these correlations are not available in UK Biobank.

With available datasets, we think it is unlikely to be feasible to accurately estimate the impact of confounding reduction and collider bias in the WSP model in empirical data. However, we believe that both our simulated and empirical data illustrate that the impact of confounding reduction and collider bias on WSP estimates could range from minimal to substantial depending on the underlying mechanism of assortative mating. This has important implications for future studies attempting to model spousal variation and studies triangulating evidence across different study designs.

Lastly, a slightly minor concern, is that the authors assumed that spouses assort on ancestry compared to non-spousal pairs (e.g. paragraph 2 of Discussion). I think this comes from the belief that ancestry are the most common confounder in genetic association studies, and there are correlations in PC space between spouses – but it is not actually demonstrated that the correlation in PC space is related to ancestry, considering that only the white British UKB individuals were used in the analysis. (And note that PCA would be sensitive to the choice of individuals used in the analysis, thus subject to all upstream ascertainment practices. In other words, demonstration would need to occur in-analysis, rather than citing publications investigating population structure in UKB in general).

We agree that principal components are sensitive to the study population and may not accurately capture ancestry. Therefore, we’ve computed spousal (and sibling) correlations for birth coordinates (north-south, east-west), an additional proxy for ancestry, and added these to Supplementary Table 4. We found strong evidence that spouses were strongly correlated for both north-south and east-west birth coordinates. Although birth coordinates are an indirect measure of ancestry, they are known to be strongly correlated with ancestry and the observed correlations illustrate how assortative mating and social homogamy can induce spousal ancestral similarities.

We have added the following to the text to describe these results and elaborate their implications for the WSP model.

Results (pages 7-8):

Before: Spouse pairs were correlated for these first 10 principal components with correlations ranging from 0.10 for PC6 to 0.32 for PC4. As expected, sibling pairs were very strongly correlated for the first 10 principal components with correlations ranging from 0.74 for PC10 to 0.98 for PC4 (**Supplementary Table 4**).

After: Spouse pairs were correlated for both birth coordinates and the first 10 principal components with correlations ranging from 0.10 for PC6 to 0.32 for PC4 across the principal components and strong correlations observed for both north-south (0.62; 95% C.I. 0.61, 0.62) and east-west (0.46; 95% C.I. 0.46, 0.47) birth coordinates. As expected, sibling pairs were very strongly correlated for birth coordinates and the first 10 principal components with correlations ranging from 0.74 for PC10 to 0.98 for PC4 (**Supplementary Table 4**). The spousal correlations for birth coordinates and principal components illustrate how assortative mating and social homogamy induce ancestral similarities between spouses.

Discussion (page 10):

Before: An example of a potential confounder in genetic association studies is ancestry, which we showed to be more correlated between spouses than for non-spouse pairs.

After: An example of a potential confounder in genetic association studies is ancestry, which we showed to be more correlated between spouses than for non-spouse pairs **by illustrating birth coordinate and principal component correlations between spouses.**
